# Impact of food insecurity and its influencing factors on the risk of malnutrition among COVID-19 patients

**DOI:** 10.1371/journal.pone.0287311

**Published:** 2023-06-15

**Authors:** Ahlam Badreldin El Shikieri

**Affiliations:** Clinical Nutrition Department, College of Applied Medical Sciences, Taibah University, Al Madinah Al Munawarah, Saudi Arabia; St John’s University, UNITED STATES

## Abstract

**Introduction:**

Few studies focused on the impact of food insecurity severity on the risk of malnutrition among COVID-19 patients in Saudi Arabia.

**Objectives:**

This study described the prevalence of food insecurity among COVID-19 patients, its severity, and its associated factors. Additionally, the impact of the severity of food insecurity on the risk of malnutrition was determined. It is hypothesized that food insecurity is associated with an increased risk of malnutrition among COVID-19 patients.

**Method:**

A cross-sectional study was conducted in Al Madinah Al Munawarah in Saudi Arabia. Patients with confirmed COVID-19 and acute severe or nonsevere illness were enrolled. The Food Insecurity Experience Scale was used to determine the severity of food insecurity, and risk of malnutrition was assessed using the Malnutrition Screening Tool. Demographic characteristics, history of medical conditions, food intake, and body mass index (BMI) were assessed.

**Results:**

A total of 514 patients were enrolled, with 391 (76%) having acute nonsevere COVID-19 symptoms. A total of 14.2% of patients suffered from food insecurity. Of these, 17% had severe symptoms. The severity of food insecurity was influenced by patients’ education (P = 0.02, 95% CI 0.019–0.225), weight loss (P = 0.0001, 95% CI 0.252–0.752), and loss of appetite (P = 0.0001, 95% CI 0.293–0.604). Fifteen percent of patients were at risk of malnutrition. Many obese patients suffered from severe COVID-19 symptoms (P = 0.029, 95% CI 0.02–0.539). The risk of malnutrition was associated with the severity of food insecurity (P = 0.001, 95% CI -0.056- -0.014), BMI (P = 0.049, 95% CI 0.000–0.042), and employment status (P = 0.034, 95% CI 0.002–0.048).

**Conclusion:**

Food insecurity and the risk of malnutrition among COVID-19 patients should be assessed to prevent adverse health outcomes.

## Introduction

Coronaviruses (CoVs) constitute a major virus family that has expanded worldwide since December 2019. Novel coronavirus is called severe acute respiratory syndrome coronavirus 2 (SARS-CoV-2) or COVID-19 [1, 2]. The COVID-19 pandemic has impaired people’s social and economic stability, health, and health care in high- and low- to middle-income countries, the latter being the most affected [3]. In addition, the pandemic has increased the prevalence of food insecurity and risk of malnutrition [4], which were already high in low- to middle-income countries.

Food insecurity (FI), defined as "*the limited or uncertain availability of nutritionally adequate and safe foods*, *or limited or uncertain ability to acquire acceptable foods in socially acceptable ways*", is a global burden [5, 6], especially after the pandemic [7]. Food instability can have a direct or indirect impact on health. It has been linked to under- and overnutrition [8], impaired mental health symptoms such as sadness and anxiety, and a worse quality of life [9–11].

During the pandemic, successive lockdowns threatened people’s access to food in low-, middle-, and high-income countries. External factors such as food availability and quality of food and personal factors such as affordability and geographical access disrupted the global food environment [12]. The disturbed food environment led to an increased prevalence of FI in high-, middle- and low-income countries, ranging from 8% in North America and Europe to 24% in Asia and 58% in Africa [13]. COVID-19 and FI have commonalities between them. Both adversely impair people’s health, well-being, and nutritional status [14].

Nutrition is considered one of the critical elements of health; it enhances the immune system [15]. Nutritional status is considered an indicator of *"resilience against destabilization*", as stated by Naja & Hamadeh in 2020 [16]. In addition, malnutrition can predict the progression of various diseases, including respiratory failure [17]. Thus, determining the risk of malnutrition among COVID-19 patients can assist dieticians and health care providers in providing appropriate medical nutrition therapy to speed up patients’ recovery rate and reduce adverse effects.

Although FI affects people’s health and nutritional status, the impact of COVID-19 on the severity of FI and its impact on the risk of malnutrition among infected people is still poorly documented, especially in high-income countries such as Saudi Arabia. A literature search revealed no published studies in Saudi Arabia or other Arab countries focusing on FI among COVID-19 patients and its impact on their nutritional status. The current paper aimed to determine the experience of food insecurity among COVID-19 patients, its severity, influencing factors, and its impact on the risk of malnutrition. It was hypothesized that many patients would be food insecure (H1). Furthermore, food insecurity and associated factors would increase the risk of malnutrition (H2).

### Materials and methods

An epidemiological, cross-sectional study was conducted to fulfil the study objectives. This study was conducted in Al Madinah Al Munawarah, located in the Hijaz Region in northwestern Saudi Arabia, 250 kilometers (160 miles) east of the Red Sea and 620 meters (2,030 feet) above sea level. Data were collected between mid-May and mid-September 2020. During that time, Al Madinah Al Munawarah, where the study was conducted, was among the cities with high rates of active COVID-19 cases.

COVID-19 patients in isolation centers and those admitted to the Ohud and General Madinah Hospitals were recruited. Only patients who were residents of Al Madinah Al Munawarah were included. Patients who were COVID-19 positive as determined by their nasal polymerase chain reaction (PCR) results and were admitted to isolation centers with acute nonsevere symptoms (e.g., cough, fever, runny nose, fatigue, headache, loss of smell and taste, and sore throat) were included. These patients did not need hospitalization due to their stable health status. In addition, patients with acute severe symptoms such as shortness of breath and mild or severe pneumonia who needed further medical assistance and were not in an intensive care unit were recruited from the Ohud and General Madinah Hospitals.

The disease duration ranged from seven days (4.0±2.2 days) in the isolation centers to 15 days (8.0±4.5 days) in the hospitals. Patients who gave their consent were included. Patients who were Arabic and/or English speakers were included to avoid communication barriers. Patients in intensive care units who needed mechanical ventilation or were not COVID-19 positive, on enteral nutrition, or had severe mental health impairments were excluded. At the time of this study, Al Madinah Al Munawarah was among the top places in the Kingdom with active cases (n = 2,634), accounting for 20.4% of total cases in Saudi Arabia (https://covid19.moh.gov.sa/ Accessed 23/04/2020).

The study sample size (n) was determined using the following formula:

$$n=Z_{1‐\alpha /2}{}^{2}p(1‐p)÷d^{2}\;where\;Z_{1‐\alpha /2}=1.96,p=the\;prevalence\;of\;active\;COVID‐19\;cases\;in\;Al\;Madinah\;Al\;Munawarah\;in\;March=20.4\%,\;d=0.05$$

After adding 10% for anticipated patient dropouts, the number of patients had to be at least 270. In addition, the outcome variables included in this study were the severity of food insecurity and impaired nutritional status. The exposures were the severity of COVID-19, demographic characteristics, medical history, weight loss, appetite loss, low dietary intake, and BMI.

The nurses who covered the hospital wards and/or the isolation centers assisted in data collection and completed a structured questionnaire with the COVID-19 patients. The following information was obtained: demographic characteristics and background information: sex (man or woman), age (20–40, 41-≥65 years; no minors <18 years were included), education level (no formal schooling, elementary or religious schooling), marital status (single, married, divorced, widowed), nationality (Saudi, non-Saudi), employment status (retired, nongovernmental employee, governmental employee, housewife), severity of the disease (acute severe, acute nonsevere), and medical history (including psychiatric illnesses). These acted as the covariates or the confounding variables of this study.

Furthermore, the Food Insecurity Experience Scale Survey Module for Individuals (FIES SM-I) was used to measure the degree of severity of food insecurity. The Arabic version of the FIES SM-I was utilized. The scale consists of eight yes/no questions and assesses the psychological components of food insecurity in a noninvasive, low-cost manner during the last 30 days. In addition, the FIES SM-I assists in determining the relationship between FI and nutritional outcomes (e.g., anthropometric measurements and risk of malnutrition) [18]. A single-parameter logistic model, the item response theory model, was utilized to estimate the severity parameters from each question response. Rasch reliability statistics were employed to assess the model assumptions of equal discrimination and overall model fit [19]. Food insecurity status was determined by adding the results from all eight items and putting them into one of four categories of food insecurity (raw score range): food secure (0), mild (1–3), moderate (4–6), and severe (7–8) [20]. The exposure variable was food insecurity.

To identify malnutrition risk, several screening methods, such as the Malnutrition Universal Screening Tool and the Mini Nutrition Assessment-Short Form, are employed in general clinical practices or specialized illness contexts [21]. The Malnutrition Screening Tool (MST) was used in our study to screen patients for the risk of malnutrition. The tool might be utilized in various contexts for adults with confirmed or suspected COVID-19, regardless of age or sex [22]. The tool works by asking two questions that are then scored. These questions were regarding changes in appetite and weight. In earlier research, the MST demonstrated strong interrater reliability (>90%), specificity (93%), and sensitivity (93%) [23]. A score of 0–1 indicated a low risk of malnutrition, a score of 2 indicated a moderate risk of malnutrition, and a score of 3–5 indicated a high risk of malnutrition. In addition, information about changes food intake was collected. The risk of malnutrition was the outcome variable.

Additionally, an electronic version of the questionnaire was used to reduce direct contact with COVID-19 patients. Furthermore, patients’ nutritional status was determined using the Quetelet body mass index (BMI), a known effective measure to define weight-related health risks [24]. BMI was determined using established formulae (weight in kilograms ÷ height in meters squared). Weight was measured twice per the approved method. The OMRON Body Fat Scale (BF508l, China) was utilized after calibration. Patients were requested to remove any heavy clothing, shoes, or accessories, and measurements were collected to the nearest 0.1 kg. Height was measured twice with a portable calibrated stadiometer (SECA-213 model, Germany). Patients were requested to remove their shoes and maintain a perpendicular line of sight. BMI was defined using the World Health Organization (WHO) categorization as severely underweight (16.5 kg/m^2^), underweight (18.5 kg/m^2^), normal weight (18.5–24.9 kg/m^2^), overweight (25.0–29.9 kg/m^2^), and obese (30 kg/m^2^) [25]. BMI was assessed on admission to isolation centers and hospitals.

Ethical permission was sought from the IRB at the College of Applied Medical Sciences, Taibah University (**SREC/AMS 2020/63/CND**). Permission was also obtained from the IRB at the Directorate of Health Affairs in Al Madinah Al Munawarah (**IRB 493**). Patients gave consent to participate before the start of this study after being assured that participation was voluntary and that all data would be treated with confidentiality and privacy. Patients who could read and write signed an informed consent form, whereas illiterate patients gave verbal approval. Patients were further informed that they had the right to withdraw from this study without any obligations or pressure.

Nurses who collected data wore personal protective equipment (PPE) before working with patients and spent 15 minutes with each patient. Afterward, nurses either returned at another time or called the patients (if they did not attend) on the telephone. Data collectors were trained before the start of the project from 7–13 May 2020 to ensure the standardization of data collection methods and reduce personal bias. In addition, the questionnaires were pretested with ten patients before the commencement of this study.

The frequency and proportion of categorical factors (sex, nationality, nutritional status, food consumption, hunger, and weight loss) were described. Standard criteria were used to assess the normality of data for continuous variables. The means and standard deviations of normally distributed continuous variables (age, height, and weight) were reported. Pearson correlation was employed to analyze the relationship between various variables. To evaluate whether there were any statistically significant differences between the means of more than two separate groups, analysis of variance (ANOVA) and Tukey’s post hoc test were performed (severity of food insecurity and risk of malnutrition). Multiple linear regression analysis and multinomial logistic regression were used to examine the extent to which a linear connection existed between a dependent variable and one or more independent variables. After controlling for confounders, the relationship between risk variables and outcomes was expressed as beta values and 95% confidence intervals (CIs). The statistical significance level was set at P<0.05. The dataset can be accessed via **https://doi.org/10.5061/dryad.5tb2rbp72**.

## Results

A total of 564 patients were surveyed, and 50 were excluded due to language barriers (n = 35) or refusal to participate (n = 15). Thus, the final number of patients who completed this study was 514. The response rate was 91.1%. The mean Rasch reliability statistic was 0.72, and the correlation between the scale items was 88.1% without intersections between the principal components of the residuals (S1 Fig). The absolute difference between country and global standard items is shown in S1 Table.

Of the 514 patients, 14.2% suffered from food insecurity (5.1% mild, 5.4% moderate, and 3.7% severe). The scale items commonly reported to be linked with food insecurity were "*Eating less than they thought they should because of a lack of money or other resources*" and "*Few kinds of foods eaten because of a lack of money or other resources"* (S2 Table).

Most patients included had acute nonsevere COVID-19, were aged <40 years, were men and had low education levels. Additionally, many patients were married, nongovernmental employees, non-Saudi, and had no history of chronic diseases (Table 1). When categorized based on the severity of their food insecurity, there were statistical differences among patients regarding the severity of the disease, marital status, employment status, weight loss, decreased food intake, and loss of appetite (Table 1). Fewer patients with nonsevere symptoms (those admitted to isolation centers) were food insecure (n = 52, 13.3%) compared to their counterparts who were hospitalized (n = 21, 17.1%). Additionally, many food-secure patients had nongovernmental jobs compared to moderately food-insecure patients (P = 0.005, CI 0.122–0.975). Regarding their employment status, moderately food-insecure patients differed statistically from mildly food-insecure patients (P = 0.002; CI 0.214–1.304).

**Table 1:** Demographic characteristics and nutritional status of COVID-19 patients as categorized by the severity of food insecurity (n = 514 patients).

Factors	Food secure	Mild FI	Moderate FI	Severe FI	Total	P value^#^
	(n = 441,85.8%)	(n = 26,5.1%)	(n = 28,5.4%)	(n = 19,3.7%)	(n = 514)	
**Severity of COVID-19**						**0.0001** ^**§**^
Acute nonsevere	339 (77)	20 (77)	22 (78.6)	10 (52.6)	391 (76)	
Acute severe	102 (23)	6 (23)	6 (21.4)	9 (47.4)	123 (24)	
**Sex**						**0.279** ^**§**^
Men	360 (82)	19 (73)	22 (78.6)	18 (94.7)	419 (81.5)	
**Age (years)**						**0.492**
<20–40	230 (52)	13 (50)	13 (46.5)	7 (37)	263 (51.2)	
41–65	211 (48)	13 (50)	15 (53.5)	12 (63)	251 (48.8)	
**Education level**						**0.528**
No schooling, elementary and religious schools	307 (70)	14 (53.8)	16 (57)	10 (52.6)	347 (67.5)	
High school	74 (17)	3 (11.5)	9 (32)	5 (26.3)	91 (17.7)	
University and above	60 (13)	9 (34.6)	3 (11)	4 (21.1)	76 (14.8)	
**Marital status**						0.049*
Married	372 (84.4)	23 (88.5)	23 (82)	17 (89.5)	435 (84.6)	
**Employment status**						0.01*
Nongovernmental employee	304 (69)	12 (46.2)	16 (57)	11 (58)	343 (66.7)	
**Nationality**						**0.058**
Non-Saudi	420 (79)	14 (53.8)	18 (68)	14 (73.7)	396 (77)	
**Medical history**						**0.571**
No history of diseases	359 (81.4)	21 (80.8)	23 (82)	7 (36.8)	410 (79.8)	
Diabetes and/or hypertension	70 (16)	2 (7.7)	23 (18)	11 (58)	78 (15.2)	
Others^#^	12 (2.6)	3 (11.5)	-	1 (5.3)	26 (5)	
**Nutrition-related symptoms**						
Weight loss	68 (15.4)	4 (15.3)	5 (18.0)	4 (21.1)	81 (15.8)	0.0001***
Decreased food intake	81 (18.4)	3 (11.5)	6 (21.4)	9 (47.4)	99 (19.3)	0.0001***
Loss of appetite	110 (25.6)	17 (44.7)	19 (68.0)	19 (100)	165 (32.1)	0.0001***
**Risk of malnutrition**						**0.994**
Low risk	377 (85.5)	22 (84.6)	24 (85.7)	14 (73.7)	437 (85.0)	
Moderate risk	60 (13.6)	3 (11.5)	3 (10.7)	4 (21.1)	70 (13.6)	
High risk	4 (0.9)	1 (3.8)	1 (3.6)	1 (5.3)	7 (1.4)	
**BMI (kg/m** ^**-2**^ **)**	**n = 424**	**n = 24**	**n = 25**	**n = 18**	**n = 491**	**0.129**
**Mean (SD)**	25.4 (5.0)	25.3 (4.1)	23.6 (4.7)	27.0 (5.3)	25.4 (5.0)	
Underweight	19 (4.3)	-	2 (7.1)	-	21 (4.3)	
Normal weight	205 (46.5)	10 (38.5)	17 (60.7)	8 (42.1)	240 (49.0)	
Overweight	131 (29.7)	10 (38.5)	3 (10.7)	6 (31.6)	150 (30.5)	
Obese^†^	69 (15.6)	4 (15.4)	3 (10.7)	4 (21.4)	80 (16.3)	

*P values based on Tukey’s HSD ANOVA; ^**§**^P values obtained from the chi-square test; ^#^Medical conditions included chronic kidney failure, asthma, epilepsy, etc.; FI = food insecurity; The FIES-SM-I was used to assess the severity of food insecurity; Values are numbers and percentages.

Furthermore, food insecurity was positively associated with weight loss (P = 0.003; CI 0.119–0.782), decreased food intake (P = 0.006; CI 0.071–0.570), and loss of appetite (P = 0.0001; CI 0.139–0.554). There were no statistical differences between normal and overweight patients. Irrespective of the severity of food insecurity, many patients were overweight/obese (Table 1).

Multinomial logistic regression revealed that the severity of FI as the dependent variable was associated with the severity of disease (P = 0.0001, χ^2^ = 29), education level (P = 0.001, χ^2^ = 22.3), employment status (P = 0.15, χ^2^ = 25), nationality (P = 0.29, χ^2^ = 23), loss of appetite (P = 0.001, χ^2^ = 17.3) and decreased food intake (P = 0.037, χ^2^ = 13.4). Among COVID-19 patients, moderate FI was associated with decreased food intake (P = 0.0001, B = 18.3). In addition, severe FI was negatively associated with the severity of disease (P = 0.02, B = -7.94), employment status (P = 0.02, B = 3.3), and loss of appetite (P = 0.039, B = -6.5).

Overall, 15% of COVID-19 patients (n = 77) had impaired nutritional status, with many having a moderate risk of malnutrition (n = 70, 13.6%) and a few having a high risk of malnutrition (n = 7, 1.4%). Eighty patients (16%) were obese, 150 (31%) were overweight, 240 (49%) had normal weight, and 21 (4%) were underweight. Patients with acute severe COVID-19 were more likely to have a higher risk of malnutrition than acute nonsevere patients, who mostly had a moderate risk of malnutrition (results not shown in table).

Malnourished patients were older (aged >41 years), had low education levels, were non-Saudi, and had a history of chronic diseases (Table 2). In addition, a previous history of diabetes and/or hypertension was positively associated with a moderate or high risk of malnutrition. The risk of malnutrition was negatively correlated with BMI. Patients with a moderate or high risk of malnutrition were overweight and/or obese and had decreased food intake, weight loss, and loss of appetite compared to their counterparts (Table 3). Additionally, a moderate or high risk of malnutrition was associated with moderate/severe food insecurity (Table 3).

**Table 2:** Demographic characteristics of COVID-19 patients as categorized by their nutritional status as determined by the MST (n = 514).

Factors	Low risk	Moderate risk	High risk	P value
	(n = 437, 85%)	(n = 70, 13.6%)	(n = 7, 1.4%)	
**Severity of COVID-19**				**0.0001** ^**§**^
Acute nonsevere	373 (85.4)	53 (75.7)	1 (14.3)	
Acute severe	64 (14.6)	17 (24.3)	6 (85.7)	
**Sex**				**0.273** ^**§**^
Men	362 (83)	52 (74.3)	5 (71.4)	
**Age (years)**				**0.0001**
<20–40	240 (55)	23 (33)	-	
41–65	197 (45)	47 (67)	7 (100)	
**Education level**				**0.0001**
No schooling, elementary and religious schools	316 (72.3)	26 (37)	5 (71.4)	
High school	62 (14.2)	28 (40)	1 (14.3)	
University and above	59 (13.5)	16 (23)	1 (14.3)	
**Marital status**				**0.321**
Married	368 (84.2)	61 (87)	6 (85.7)	
**Employment status**				**0.403**
Nongovernmental employee	313 (71.6)	27 (38.6)	3 (43)	
**Nationality**				**0.0001**
Non-Saudi	362 (83)	30 (43)	4 (57)	
**Medical History**				**0.0001**
No history of diseases	361 (82.6)	46 (65.7)	1 (14.3)	
Diabetes and/or hypertension	55 (12.6)	18 (25.7)	5 (71.4)	
Others^#^	21 (4.8)	6 (8.6)	1 (14.3)	

^#^Medical conditions included chronic kidney failure, asthma, epilepsy, etc. Values are numbers and percentages. *P values based on ANOVA with Tukey’s HSD; ^**§**^P values obtained using the chi-square test

**Table 3:** Nutrition-related factors and the severity of food insecurity among COVID-19 patients as categorized by their nutritional status as determined by the MST (n = 514 patients).

Factors	Low risk	Moderate risk	High risk	P value
	(n = 437, 85%)	(n = 70, 13.6%)	(n = 7, 1.4%)	
**BMI (kg/m** ^**-2**^ **)**	**n = 418**	**n = 66**	**n = 7**	**0.0001**
**Mean (SD)**	25.0 (4.6)	29.0 (5.6)	24.6 (4.2)	
Underweight	21 (4.8)	-	-	
Normal weight	220 (50.3)	16 (23)	4 (57.1)	
Overweight	123 (28.1)	25 (35.7)	2 (28.6)	
Obese^†^	54 (12.4)	25 (35.7)	1 (14.3)	
**Nutrition-related symptoms**				
Decreased food intake	40 (9.2)	54 (77.1)	5 (71.4)	**0.0001**
Weight loss	8 (1.8)	66 (94.3)	7 (100)	**0.0001**
Loss of appetite	89 (20.4)	69 (98.6)	7 (100)	**0.0001**
**Severity of food insecurity**				**0.017**
Food secure	377 (86.3)	60 (85.7)	4 (57.1)	
Mild insecurity	22 (5)	3 (4.3)	1 (14.3)	
Moderate insecurity	25 (5.5)	3 (4.3)	1 (14.3)	
Severe insecurity	14 (3.2)	4 (5.7)	1 (14.3)	

*P values based on ANOVA with Tukey’s HSD. The risk of malnutrition was determined using the MST.

Furthermore, when categorized according to BMI, statistical differences were found in the severity of COVID-19 (severe or nonsevere), sex, age, education level, marital status, employment status, and nationality. There were statistical differences in medical history, food intake, weight loss, and loss of appetite based on BMI categories (Table 4). For instance, severe COVID-19 symptoms were more often associated with overweight (P = 0.043, CI 0.034–0.254) and underweight (P = 0.029, CI 0.02–0.539).

**Table 4:** Demographic characteristics, nutritional factors, and the severity of food insecurity of COVID-19 patients as categorized by their BMI (n = 491).

Factors	Obese	Overweight	Normal weight	Underweight	P value
	(n = 80, 16%)	(n = 150, 31%)	(n = 240, 49%)	(n = 21, 4)%	
**Severity of COVID-19**					**0.0001** ^**§**^
Acute nonsevere	50 (62.5)	106 (77.7)	205 (85)	19 (90.5)	
Acute severe	30 (37.5)	44 (29.3)	35 (15)	2 (9.5)	
**Sex**					**0.002** ^**§**^
Men	58 (72.5)	118 (78.7)	214 (88.8)	15 (71.4)	
**Age (years)**					
<20–40	32 (40)	60 (40)	147 (61.3)	15 (71.4)	
41–65	48 (60)	90 (60)	93 (38.7)	6 (28.6)	
**Education level**					**0.003**
No schooling, elementary and religious schools	45 (56)	95 (63.3)	180 (75)	16 (76)	
High school	17 (21)	32 (21.3)	37 (15)	4 (19)	
University and above	18 (23)	23 (15.4)	23 (10)	1 (5)	
**Marital status**					**0.01**
Married	70 (87.5)	133 (88.7)	201 (83)	15 (71.4)	
**Employment status**					**0.006**
Nongovernmental employee	45 (56.3)	97 (65)	184 (76)	13 (62)	
**Nationality**					**0.03**
Non-Saudi	48 (60)	108 (72)	206 (85.8)	15 (71.4)	
**Medical History**					**0.005**
No history of diseases	60 (75)	112 (74.6)	205 (85)	21 (100)	
Diabetes and/or hypertension	16 (21.2)	28 (18.7)	25 (10)	-	
Others^#^	4 (3.8)	10 (6.7)	10 (4)	-	
**Nutrition-related symptoms**					
Decreased food intake	28 (35)	36 (24)	25 (10.4)	1 (4.8)	**0.0001**
Weight loss	28 (35)	27 (18)	22 (9.2)	**-**	**0.004**
Loss of appetite	44 (55)	55 (36.7)	52 (21.6)	5 (23.8)	**0.0001**
**Risk of malnutrition**					**0.89**
Low risk	54 (67.5)	123 (82)	220 (91.3)	21 (100)	
Moderate risk	25 (31.3)	25 (16.7)	16 (6.6)	-	
High risk	1 (1.3)	2 (1.3)	4 (2.1)	-	
**Severity of food insecurity**					**0.11**
Food secure	67 (83.8)	117 (78)	205 (85)	20 (95)	
Mild insecurity	2 (2.4)	16 (10.7)	20 (8.3)	-	
Moderate insecurity	7 (8.8)	11 (7.3)	7 (3)	1 (4.8)	
Severe insecurity	4 (5)	6 (4)	9 (3.7)	-	

^#^Medical conditions included chronic kidney failure, asthma, epilepsy, etc. Values are numbers and percentages. *P values based on ANOVA with Tukey’s HSD; ^**§**^P values obtained using the chi-square test

Furthermore, many patients with low education levels had normal BMIs, whereas most obese patients had university degrees (P = 0.004, CI 0.073–0.546). Moreover, many overweight patients had a history of chronic diseases compared with their normal-weight counterparts (P = 0.027, CI 0.029–0.706). Additionally, overweight and obesity were associated with weight loss, decreased food intake, and loss of appetite.

Moreover, multiple linear regression revealed that the severity of food insecurity (the dependent variable) was influenced by the patient’s education level, weight loss, and loss of appetite (S3 Table). Additionally, multiple linear regression showed that the patient’s risk of malnutrition (the dependent variable) was mainly influenced by the severity of COVID-19, employment status, nationality, weight loss, BMI, and the severity of food insecurity (Table 5).

**Table 5:** Multiple linear regression model for the risk of malnutrition and its determinants.

	B	Std. Error	Beta	t	Significance level	95% CI	
Severity of COVID-19	0.104	0.023	0.107	4.411	0.0001***	0.057	0.150
Sex	0.005	0.024	0.005	0.214	0.831	-0.043	0.053
Age	0.011	0.009	0.029	1.255	0.210	-0.006	0.029
Education level	0.008	0.012	0.014	0.632	0.528	-0.016	0.032
Marital status	0.010	0.023	0.009	0.451	0.653	-0.035	0.056
Employment status	0.025	0.012	0.051	2.130	0.034*	0.002	0.048
Nationality	-0.018	0.006	-0.066	-2.991	0.003**	-0.029	-0.006
Medical history	0.009	0.007	0.027	1.281	0.201	-0.005	0.022
Weight loss	0.558	0.017	0.759	33.569	0.0001***	0.525	0.591
Decreased food intake	0.122	0.022	0.129	5.576	0.0001***	0.079	0.166
BMI	0.021	0.011	0.042	1.976	0.049*	0.000	0.042
Severity of food insecurity	-0.035	0.011	-0.064	-3.267	0.001**	-0.056	-0.014

*P<0.01, **P<0.001, ***P<0.0001. The independent variables entered in the model were severity of food insecurity, employment status, marital status, medical history, BMI, education level, nationality, age, sex, decreased food intake, severity of COVID-19, and weight loss. The risk of malnutrition was the dependent variable. Multiple regression model: R = 0.9, R^2^ = 0.811, adjusted R^2^ = 0.804, F = 124.919, significant F change = 0.000

## Discussion

COVID-19 is among the leading causes of morbidity and mortality worldwide. Moreover, lockdowns and social distancing during the early months of the pandemic disrupted the food security status of individuals and households in both low-middle- and high-income countries. Unfortunately, several countries still suffer from a high prevalence of food insecurity, which impairs the population’s health and nutritional status [26]. Our study aimed to determine the prevalence of food insecurity among COVID-19 patients and its influential factors. The current study revealed that the prevalence of food insecurity was high, affecting 14% of the patients mostly because they did not have enough money to buy food, which led to buying little food or eating less. Thus, this study’s hypothesis that many patients suffer from food insecurity was confirmed. Compared with Iranian patients, the prevalence in our study was higher (10% versus 14%, respectively) [27].

The current study also showed that patients’ employment status impacted food insecurity. The pandemic led to an economic downturn in many countries, including Saudi Arabia. As a result, there was a decrease in job creation, with many people becoming jobless and losing all their income or part of it [28]. Similar findings confirmed that the pandemic affected people’s employment status [6]. The pandemic affected most primary, secondary and tertiary sectors [29]. In their study, Miner and colleagues found that unemployment among emergency department patients was associated with hunger and food insecurity [30].

On the one hand, our study revealed that education level was associated with the severity of food insecurity among COVID-19 patients (based on the multiple linear regression model). Low education levels were prevalent among moderate and severe food-insecure patients. Our findings agreed with previously published results in which patients with diplomas suffered greater food insecurity than their counterparts [31]. Education, which is a measure of human capital, is correlated with productivity, efficiency, increased income, improved health and better decision-making [32]. Those with a better education have less disrupted food availability and better income-generating activities [33].

On the other hand, we did not find any relationship between food insecurity and overweight/obesity. As previously reported in other studies, food insecurity was prevalent among all BMI groups [9, 31]. This result could support the general findings regarding weight status of the adult population in Saudi Arabia, which has a high prevalence of overweight/obesity.

Moreover, impaired immunity is a risk factor for infection leading to respiratory diseases such as COVID-19. At the same time, proper nutrition is associated with a strengthened immune system that can fight the consequences of infections [34, 35]. Our findings were in accordance with those of other studies that reported that patients with a higher malnutrition risk were those with severe disease symptoms (acute severe patients) [34–36]. Moreover, our study’s prevalence of a moderate risk of malnutrition (24.3%) was lower than that among hospitalized French COVID-19 patients (49%). However, more hospitalized patients (those with severe acute symptoms) were at high risk of malnutrition (85.7%) than their French counterparts (35.7%) [37]. The authors of the study conducted at the E3M Institute in France revealed a similar prevalence of patients with a moderate risk of malnutrition (23.7% versus 24.3%) and a lower number of patients with a high risk of malnutrition (18.4% versus 85.7%) [38]. Caution should be taken when comparing the prevalence of the risk of malnutrition in studies due to the various screening tools used. For instance, the nutrition risk index, which includes serum albumin levels, was used in the French study [37], whereas we used the MST. In their study, Bedrock and colleagues assessed malnutrition using the Global Leadership Initiative on Malnutrition [38].

Consistent with our study findings, malnutrition among French [31] and Chinese [39] patients was associated with age; many patients aged >41 years were malnourished. However, the nutritional status of patients admitted to the E3M Institute in France was not associated with age [38]. Moreover, the current study revealed that the risk of malnutrition was associated with BMI, history of chronic diseases, decreased food intake, weight loss, and loss of appetite. Weight loss was previously reported as one of the most critical risk factors for malnutrition among COVID-19 patients [40]. Similar to our findings, the nutritional status of French patients differed according to their weight loss [38]. Contrary to our findings, the food intake of French COVID-19 patients was not associated with malnutrition [37].

Unlike our study findings, high rather than low BMI is associated with malnutrition risk [37]. In our study, obese patients were more likely to have severe symptoms. Similar to the findings in our study, overweight Chinese patients, diabetic patients, and cardiovascular disease patients were more likely to develop unfavorable clinical outcomes in a study [39–41]. Thus, obesity can be an independent risk factor for more complications among COVID-19 patients.

Food insecurity is a well-known potent stressor that negatively influences health. A prior study found a link between food insecurity, infection, and poor health outcomes [43]. The multiple regression model demonstrated that the intensity of food insecurity had a detrimental impact on the nutritional condition of COVID-19 patients. In our study, the two FIES elements most strongly associated with the degree of food insecurity were “eating less than they should because of a lack of resources” and “consuming a limited variety of meals.” Not eating enough during the previous month (the duration assessed by the FIES) could affect eating behaviors, food choices, and availability of the essential nutrients these patients need. Hence, as stated in other studies, food insecurity contributed to the unfavorable illness outcome presenting as impaired nutritional status [43].

Unfortunately, there has been no research that focuses on the severity of food insecurity among COVID-19 patients or the relationship between food insecurity and the risk of malnutrition. Food instability has been linked to an increased risk of malnutrition among HIV/AIDS patients [40–42]. Furthermore, food insecurity is related to worse nutritional and health status among American older individuals [43].

The current study had some limitations that need to be considered. We did not assess other health outcomes associated with COVID-19 and impaired nutritional status. This would have given an overview of the consequences of malnutrition, such as nutrition-related biomarkers (e.g., organ function). The cross-sectional nature of the study design has its drawbacks and potential biases, and thus findings should be interpreted with caution. Ideally, it would have been more appropriate to conduct a longitudinal study to follow up with patients and report the actual risks for developing malnutrition risk factors and health-related outcomes. Additionally, this study did not assess the effect of food insecurity on the progression of COVID-19.

Although this study had some limitations, it is considered the only study in Saudi Arabia to focus on food insecurity and its impact on patients’ nutritional status. Additionally, this study depended on valid, previously published tools, making it easy to compare findings with other studies (e.g., FIES, MST). The use of the MST and BMI as methods for assessing the nutritional status of patients increased the understanding of malnutrition risk instead of relying on the MST alone. Responses to questions were obtained mainly through face-to-face interviews with patients at their convenience. Additionally, the sample size was within the calculated limits, which increased the power of this study.

## Conclusion

Our study revealed that many patients suffered from food insecurity. The factors associated with food insecurity included the severity of COVID-19 (acute severe or acute nonsevere symptoms), marital and employment status, weight loss, decreased food intake, and loss of appetite. In addition, the nutritional status of COVID-19 patients was influenced by the severity of COVID-19, employment status, nationality, weight loss, BMI, and severity of food insecurity. Health care professionals, policy-makers, and program administrators are charged with improving health and well-being. Therefore, it is recommended that patients be assessed for their food insecurity and malnutrition risk. This assessment can help to tackle possible adverse health outcomes without delay and develop appropriate intervention programs.

## Supporting information

S1 Fig Screen plot of principal component analysis on residuals. There was no significant correlation between the residuals. The mean Rasch reliability statistic was 0.72, and the correlation between the scale items was 88.1%.
(DOCX)

S1 Table Absolute difference between Saudi Arabian’s COVID 19 patients and the global standard items. (DOCX)

S2 Table Prevalence of the components of the Food Insecurity Experience Scale among COVID-19 patients (n = 514). (DOCX)

S3 Table Multiple regression model. The severity of food insecurity and its associated factors.
(DOCX)
