# Supplementary material for: Impact of food insecurity and its influencing factors on the risk of malnutrition among COVID-19 patients
Source: PLoS One. 2023 Jun 15;18(6):e0287311. doi: 10.1371/journal.pone.0287311 (PMC10270634; doi:10.1371/journal.pone.0287311)
Supplement: S1 Fig — There was no significant correlation between the residuals. The mean Rasch reliability statistic was 0.72, and the correlation between the scale items was 88.1%. (DOCX) [file pone.0287311.s001.docx]

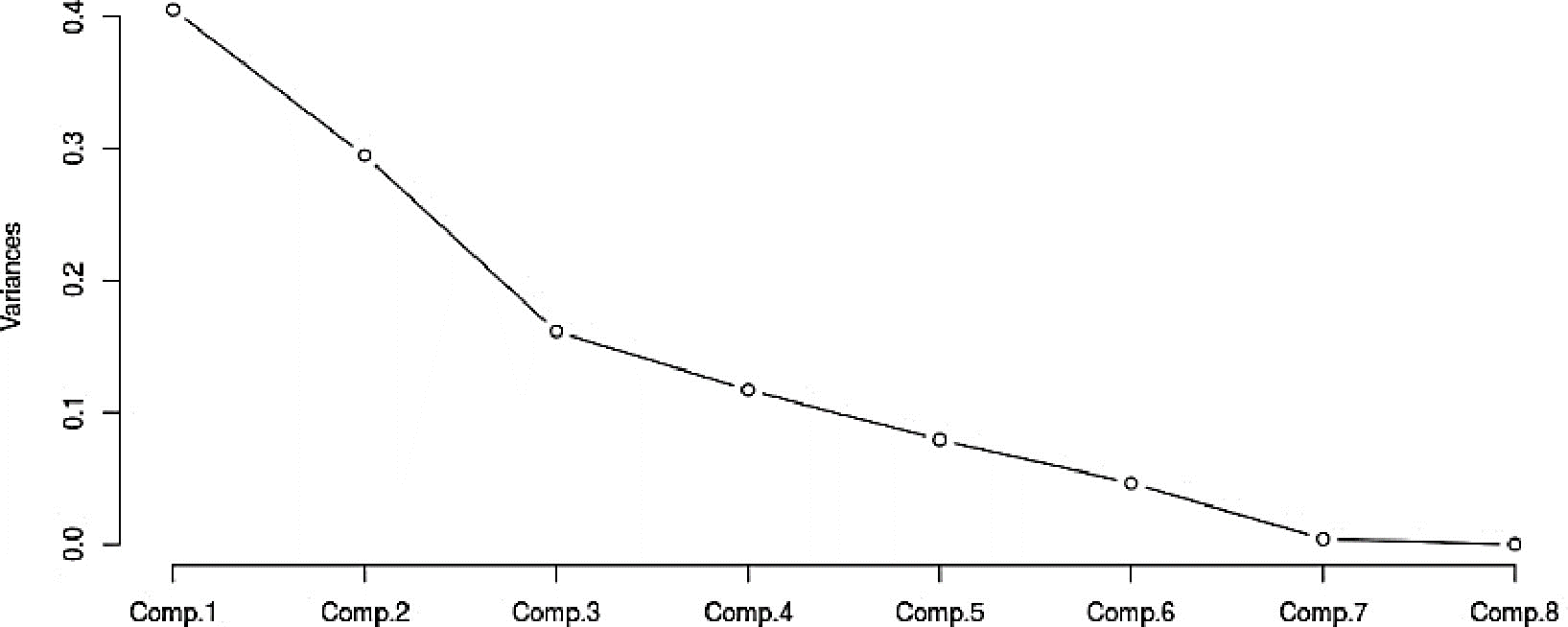


***S1 Fig:* Screen plot of principal component analysis on residuals. There was no significant correlation between the residuals.** **The mean Rasch reliability statistic was 0.72, and the correlation between the scale items was 88.1%.**
