# Supplementary material for: Impact of food insecurity and its influencing factors on the risk of malnutrition among COVID-19 patients
Source: PLoS One. 2023 Jun 15;18(6):e0287311. doi: 10.1371/journal.pone.0287311 (PMC10270634; doi:10.1371/journal.pone.0287311)
Supplement: S1 Table — (DOCX) [file pone.0287311.s002.docx]

| **S1 Table:** **Absolute difference between Saudi Arabian's COVID 19 patients and the Global standard items** | |
| --- | --- |
| Worried | 0.43 |
| Healthy | 0.66 |
| Few foods | 0.11 |
| Skipped meals | 0.88 |
| Ate less | 0.68 |
| Ran out of food | 0.16 |
| Hungry | 0.07 |
| A whole day without food | 0.12 |
