# Supplementary material for: Impact of food insecurity and its influencing factors on the risk of malnutrition among COVID-19 patients
Source: PLoS One. 2023 Jun 15;18(6):e0287311. doi: 10.1371/journal.pone.0287311 (PMC10270634; doi:10.1371/journal.pone.0287311)
Supplement: S2 Table — (DOCX) [file pone.0287311.s003.docx]

| **S2 Table: Prevalence of the components of the Food Insecurity Experience Scale among COVID-19 patients (n = 514)^#^** | |
| --- | --- |
| Worried | 55 (10.7) |
| Healthy | 47 (9.1) |
| Few foods | 58 (11.3) |
| Skipped meals | 51 (10) |
| Ate less | 58 (11.3) |
| Ran out of food | 34 (6.6) |
| Hungry | 32 (6.2) |
| A whole day without food | 21 (4.1) |

*^#^Numbers and percentages of patients who reported experiencing each of the components of the FIES assessed. The prevalence of food insecurity was 14.2%.*
