# Supplementary material for: Impact of food insecurity and its influencing factors on the risk of malnutrition among COVID-19 patients
Source: PLoS One. 2023 Jun 15;18(6):e0287311. doi: 10.1371/journal.pone.0287311 (PMC10270634; doi:10.1371/journal.pone.0287311)
Supplement: S3 Table — The severity of food insecurity and its associated factors. (DOCX) [file pone.0287311.s004.docx]

| **S3 Table: Multiple Regression Model. The severity of food insecurity and its associated factors** | | | | | | | |
| --- | --- | --- | --- | --- | --- | --- | --- |
|  | B | Std. Error | Beta | t | Significance level | 95% CI | |
| Severity of disease | 0.014 | 0.104 | 0.008 | 0.136 | 0.892 | -0.190 | 0.218 |
| Sex | 0.078 | 0.104 | 0.040 | 0.753 | 0.452 | -0.126 | 0.282 |
| Age | 0.026 | 0.038 | 0.036 | 0.676 | 0.499 | -0.049 | 0.101 |
| Education level | 0.122 | 0.052 | 0.117 | 2.331 | **0.020*** | 0.019 | 0.225 |
| Marital status | 0.064 | 0.098 | 0.031 | 0.652 | 0.515 | -0.129 | 0.257 |
| Employment status | -0.027 | 0.050 | -0.030 | -0.534 | 0.593 | -0.125 | 0.072 |
| Nationality | -0.040 | 0.025 | -0.081 | -1.573 | 0.116 | -0.090 | 0.010 |
| Medical history | 0.023 | 0.028 | 0.039 | 0.805 | 0.421 | -0.033 | 0.079 |
| Weight loss | 0.502 | 0.127 | 0.369 | 3.950 | **0.0001**** | 0.252 | 0.752 |
| Decreased food intake | 0.020 | 0.099 | 0.011 | 0.201 | 0.840 | -0.174 | 0.214 |
| Loss of appetite | 0.448 | 0.790 | 0.242 | 5.655 | **0.0001**** | 0.293 | 0.604 |

**P<0.01, **P<0.0001; The independent variables entered in the model were: nutritional status, employment status, marital status, medical history, BMI, education level, nationality, age, sex, decreased food intake, the severity of the disease, and weight loss. The severity of food insecurity was the dependent variable. Multiple Regression Model: R = 0.261, R^2^ = 0.068, Adjusted R^2^ = 0.039, F = 2.314, Significant F change = 0.003*
